# Medication management of COVID-19 patients during transition to virtual models of care: a qualitative study

**DOI:** 10.1186/s40545-023-00633-1

**Published:** 2023-10-25

**Authors:** H. Laetitia Hattingh, Catherine Edmunds, Brigid M. Gillespie

**Affiliations:** 1 Allied Health Research, Gold Coast Health, Gold Coast, QLD 4215 Australia; 2 School of Pharmacy and Medical Sciences, Griffith University, Gold Coast, QLD 4222 Australia; 3 School of Pharmacy, The University of Queensland, Brisbane, QLD 4102 Australia; 4 Medical Services, Clinical Governance and Research, Gold Coast Health, Gold Coast, QLD 4215 Australia; 5 NHMRC Wiser Wounds CRE, MHIQ, Griffith University, Gold Coast, QLD 4222 Australia; 6 Nursing and Midwifery Education and Research Unit, Gold Coast Health, Gold Coast, QLD 4215 Australia

**Keywords:** Virtual ward, Medication management, Patient safety, COVID-19 model of care

## Abstract

**Introduction:**

Expansion of hospital service models was one of the strategies implemented to manage the COVID-19 pandemic through virtual models of care. COVID-19 patients were hospital inpatients transferred to virtual wards and managed outside the hospital. Pharmacists had to provide distance medication management and support services. Virtual care patient support incorporated telehealth consultations by doctors, pharmacists and nurses. This study explored hospital clinicians’ experiences and perspectives on medication management and safety issues of the COVID-19 patients transferred from inpatient units (IPUs) to virtual models of care at the time of transfer.

**Methods:**

Semi-structured qualitative interviews were conducted with purposively selected doctors, pharmacists and nurses involved in the management of COVID-19 patients in a virtual model of care (home or hotel). Clinicians were interviewed face-to-face or via MS Teams between March and May 2022. An interview schedule included 13 questions and prompts to explore perceptions of medication management and safety aspects.

**Results:**

Twenty clinicians were interviewed: six doctors, seven pharmacists, and seven nurses. The average interview time was 26 min (SD: 4.7; range 21–39). Four major medication management and safety themes emerged from the data: (1) complexities involved in efficient handover between IPU and virtual models of care; (2) lack of clarity on roles and responsibilities between hospital and primary care clinicians; (3) communication challenges when pharmacists work remotely; and (4) proactive management of specific medication safety risks. A common thread throughout the themes was concerns for potential impact on patient safety.

**Conclusion:**

Overall, clinicians were supportive of the virtual models although patient safety issues were raised that need to be addressed in the development of future services. The results from this study may inform improvements in medication safety implementation of future virtual models of care.

## Introduction

Due to the rise in the number of individuals who contracted COVID-19, hospitals experienced a surge in patient admissions. Consequently, novel post-acute care approaches were needed which incorporated collaborative self-care. Hospitals had to reorientate healthcare services and implement models of care to cope with increased service demand, e.g. emergency department avoidance for high-risk patients [1–4]. Other options included increased implementation of digital health to provide virtual care, e.g. video consultations and telemedicine [5–8] with the COVID-19 pandemic referred to as health care’s digital revolution [9]. Hospital-at-Home models were also introduced for patients who were deemed to be at lower risk but still required monitoring [10]. These models of care provided advantages with regard to the need for physical distancing to reduce exposure risk [11, 12].

The Gold Coast Hospital and Health Service (GCHHS), Queensland, implemented new models of care to manage the demands on the healthcare and ease inpatient bed pressures [13, 14]. However, the opening of the state and territory borders in December 2021, following an extended period of interstate restrictions, resulted in a surge of the virus in Queensland, Australia. Additional models of care had to be developed which included a virtual ward with implementation of the model including patients located either at home or in a hotel environment [Hospital In The Hotel (HITHot)]. COVID-19 patients who were admitted to hospital but who required low-level ongoing care or who needed to complete isolation periods with minimal follow-up were transferred to these virtual models of care. Although these models improved access to healthcare services, assisted patients with isolation requirements and those who were interstate or homeless [15, 16], there were concerns regarding potentially lower quality of care if the requisite technology is not available or fragmented care if patients are not connected with their primary care providers [17]. There were also concerns about patients’ medication management when they transferred to the virtual model of care as hospital pharmacists were not able to provide face-to-face medicine reconciliation or review services due to the isolation requirements for COVID-19 patients.

Transitions of care, such as discharge from hospital or movement of patients within a hospital or between different health care providers, are high-risk periods for medication-related harm (MRH) [18–22]. A systematic review highlighted contributing factors to MRH during transition of care that included polypharmacy and poor quality discharge medication information [23]. COVID-19 patients who transferred from hospital to a virtual model of care could therefore have been at risk of potential MRH if appropriate processes and procedures were not in place to manage patients’ medicines and transfer of medicine information to primary care clinicians.

Studies have evaluated virtual medication management services [24–26], however there was a gap in the literature on the experiences of clinicians regarding patients’ medication management and safety when transferred to virtual models of care. A need was identified to evaluate the medication management of patients who were transferred from inpatient units (IPUs) to virtual models of care from the perspectives of the clinicians involved at the point of transfer.

## Methods

### Study design

A qualitative study was undertaken using semi-structured interviews with hospital clinicians. The consolidated criteria for reporting qualitative research (COREQ) checklist [27] and qualitative research criteria were used during the development, analysis, and reporting of the interviews [28, 29]. This study was part of a mixed-methods research project that incorporated patient surveys and a retrospective audit of medical records, reported elsewhere [30]. The project was approved by the Gold Coast Hospital and Health Service (GCHHS) Human Research Ethics Committee (LNR/2022/QGC/83951).

In this qualitative study, we used thematic analysis to identify and explore key factors that impacted on patients’ medication management.

### Aim

The aim of this study was to explore clinicians’ experiences and perspectives on the medication management of COVID-19 patients who transferred from an IPU to a virtual model of care (home or hotel).

### Setting

Data were collected at GCHHS, incorporating the Gold Coast University Hospital (GCUH), a tertiary hospital (approx. 750 beds) and Robina Hospital, a major regional hospital in the same district (approx. 350 beds). During the surge of the pandemic towards the end of 2021 there were four IPUs at GCUH and one IPU at Robina Hospital allocated to COVID-19 patients. Virtual models of care were implemented at both hospitals to reduce IPU pressure that included managing patients at home or in a hotel (HITHot service) following transition from an IPU. The virtual models were implemented in a staged approach throughout December 2021 and January 2022 as demands for health services increased.

Patients who required ongoing low-level care or needed to complete isolation periods with minimal follow-up were transferred from IPUs to these virtual models of care. Virtual models of care patients were classified as inpatients, but admitted to virtual IPUs and managed outside the hospital. During patients’ hospital stay, pharmacists assigned to IPUs were required to work remotely and conduct medication history-taking and counselling with patients over the phone. IPU pharmacists organised supply of sufficient medicine quantities before patients transferred to virtual models of care.

### Participants

We purposively selected GCUH and Robina Hospital clinicians (doctors, nurses and pharmacists) who managed COVID-19 patients who were transferred to virtual models of care. Recruitment involved purposive sampling of clinicians rostered to the COVID-19 IPUs as well as snowballing by asking participants to identify other potential participants. The aim to capture a variety of experience levels, ages and genders with approx. equal numbers of the three health professional groups. Purposive selection of potential participants allowed for maximum variation in the sampling to include participants with variation in age, gender, roles and experience, whist enabling in-depth inquiry into the topic of interest [31]. This approach provides information-rich data and improves the reliability and credibility of research findings [29].

Clinicians were invited by phone call or email between March and May 2022. Respondents were provided with a participant information and consent form detailing the study and what participation involved. Potential participants were given time (≥ 1 day) to consider their involvement.

Individual informed consent was sought from all clinicians who agreed to participate in the semi-structured interviews. Semi-structured interviews were conducted in person or via MS Teams and participant demographic information was collected before interviews commenced. All interviews were conducted by the same experienced interviewer (LH) to ensure consistency. Interviews were audio-recorded and supplemented with field notes handwritten by the interviewer. Audio recordings were transcribed verbatim, de-identified, and quality checked. Interview participants had the opportunity to check their transcripts.

Considering studies exploring the number of interviews required, the purpose of the study and the research team’s experiences, the aim was to interview 15–25 participants to reach data sufficiency [32–34].

### Interview tool

A tool to guide the semi-structured interviews was developed considering the literature [35] and team members’ expertise. The tool utilised both pre-determined open questions and the opportunity for the interviewer to explore particular themes or responses further and adapt questions as conversations progressed to allow for exploration of new ideas and concepts that participants identified as interviews progressed. It consisted of 13 questions with prompts to explore participants’ experiences and opinions about the medication management of patients who transferred to virtual models of care. The interview tool was tested for face and content validity through feedback from a researcher with expertise in qualitative interviews and three clinicians. Only minor changes were required to improve the flow of the questions.

### Data analysis

Data collection and analysis were conducted concurrently, allowing for adaptation of the semi-structured interview guide questions with emerging insights. Data were analysed using inductive content analysis to identify themes [29]. The qualitative data collection and analysis process followed an iterative approach that involved data familiarisation and researcher reflexivity. Development of initial codes incorporated triangulation of various data sources (i.e. field notes, transcripts) and subsequent identification of potential themes. Themes were reviewed to reflect on definitions and names/descriptions.

Qualitative rigor was applied by following a consistent data collection and analysis process to achieve credibility, transferability, dependability, and confirmability [36]. Steps followed included the interviewer (LH) making field notes during interviews, reflecting on interviews before sending a summary of findings to team members after each interview for input. Field notes and contact summaries were used with interview transcripts during data analysis. The initial coding framework was developed by LH and discussed with the rest of the research team (CE, BG). The team met regularly to discuss and review the coding and agreed on the coding and thematic analysis. NVivo (QSR International Pty Ltd) was used to facilitate data organisation, coding, and analysis.

## Results

### Participant characteristics

Twenty clinicians were interviewed between March and May 2022: six doctors, seven pharmacists and seven nurses. One interview was with two nurses simultaneously due to shift requirements, whereas all other interviews were one-on-one. Thirteen interviews were face-to-face and six via MS Teams. Mean interview time was 26 min (SD: 4.7; range 21–39). Participants included early career doctors with most of the other health professionals having more than 10 years’ experience in their roles. Table 1 summarises participants’ demographic data.

**Table 1:** Participant demographic data

Professional group	Male	Female	Years’ experience in current role				
			< 1	1–3	3–6	6–10	> 10
Doctor (*n* = 6)	5	1	–	1	3	1	1
Pharmacist (*n* = 7)	3	4		1	–	1	5
Nurse (*n* = 7)	1	6	–	–	–	–	7

### Themes

Four overarching medication management themes were identified through the inductive analysis. A common thread throughout the themes was concerns for the potential impact on patient safety when transferring patients to virtual models of care. Table 2 provides a summary of the themes and sub-themes. Example quotations are used throughout to contextualise the findings with the following identifiers: doctor (D), pharmacist (P) and nurse (N).

**Table 2:** Themes and sub-themes from thematic analysis

Themes	Sub-themes
Complexities involved in efficient handover between IPU and virtual models of care	Patient criteria for suitability to be transferred to virtual care
	Ongoing medicine supply considerations as patients had to isolate
	Medicine information handover challenges
Lack of clarity on roles and responsibilities between hospital and primary care clinicians	Uncertainty about responsibilities for patients’ ongoing care
	Uncertainty about responsibilities for management of patients’ chronic conditions
Communication challenges when pharmacists work remotely	Pharmacists’ challenges with contacting patients over the phone
	Additional pressure on nursing staff due to role substitution
	Decreased medication management support for doctors
Proactive management of specific medication safety risks	The need to prioritise patients with potential risk for medication-related harm
	Challenges with management of new COVID-19 therapies
	Risks of errors due to pharmacists not checking bedside lockers

### Complexities involved in efficient handover between IPU and virtual models of care

Participants agreed that the virtual models of care decreased IPU bed pressure and enabled hospitals to increase capacity and cope with large numbers of COVID-19 patients. Although the electronic medical record system facilitated continuity of care, the new models of care had certain complexities that clinicians had to overcome. Doctor participants described the various criteria that had to be considered to determine whether a patient was suitable for transfer to a virtual model of care. Certain criteria were easy to follow, e.g. patients should not require oxygen or intravenous antibiotics. However, other criteria were less rigid and required a level of clinical judgement such as close medical monitoring requirements:

*There was an impression…… they wouldn’t need immediate management….. they wouldn’t need any sort of immediate management, they’d be able to manage at home. But maybe just needed that phone call to say ‘Hey, how are things going, if anything goes backwards we can bring you back’.* P16-D

Doctors reported that they used similar criteria as those that were already in place for hospital-in-the-home (HITH) patients but in contrast with HITH, these patients were not visited by hospital staff in person. Patients’ social situations were also considered:

*[to] ensure they’ve got a safe home environment would be the first starting point … a good question is: are they living by themselves….. are they mobile enough to do the things that they need to do at home if they are going to be isolating? Are they functional enough to shower, bath, eat, administer their own medications?* P16-D

Clinicians had to navigate through various issues during the transfer process which was complicated by patients’ isolation requirements. Pharmacists highlighted specific challenges with managing patients who required medicines for chronic conditions as this was not consistently considered by the doctors and patients were not able to visit a community pharmacy for more supplies. Dispensing of medicines for chronic conditions in some cases had to be organised with the hospital dispensary:

*… the biggest issue was always the teams weren’t thinking about any of their other drugs. If they had enough supply or someone to drop it off. … The extra step was talking to the patients to make sure they had their own medications. A few times nurses would say about the script ‘they'll take the script outside’ and I said, ‘but they’re COVID positive they can’t go outside’…… redirecting the nurses as well, saying ‘No, we have to dispense it here’*. P2-P

Organising dispensing by the hospital dispensary was also a consideration for elderly patients who had to pay the full amount for non-Pharmaceutical Benefits Scheme medicines at a community pharmacy. Participants were concerned about the cost impact on patients’ while realising the extra pressure this placed on dispensary staff but judged it the best option from a patient-safety perspective:

*... at times, the safest thing to do was to supply medications from hospital from us, here at the pharmacy…… the drawback of that is the increased workload on the dispensary and pharmacy staff, but it just felt like the safest way to do things at that time*. P6-P

Pharmacist participants commented on difficulties in organising medicine information handover and quoted various instances where patients did not receive the discharge medication records (DMRs). They were concerned about the lack of handover to patients’ community pharmacies:

*It was the anticoagulant patient … I actually did phone up the patient, a couple of days later, I just wanted to make sure that they got the information and knew what they were doing with their medications. It turns out the DMR that I supplied never made it to the patient.* P6-P

*I [said] ‘do not give them to the patient if they're going to be discharged. Pharmacy needs to get involved’ and I checked the following Monday and note from the doctor: ‘patient ready for discharge, pharmacy, meds in room, patient given meds, patient discharged and they left with’. They were on about 10 or 12 meds. One was an anti-antiviral full box. No hand-over to the community pharmacy*. P1-P

### Lack of clarity on roles and responsibilities between hospital and primary care clinicians

Participants identified a need for more clearly defined roles between hospital and primary care clinicians for patients with chronic conditions. This was specifically about the ongoing management of patients’ medicines with varied opinions about who were responsible to write and organise prescriptions. Some participants argued that patients in virtual care models were classified as inpatients and their care was therefore the responsibility of hospital clinicians:

*I can see how people feel different or there’d be that slight maybe confusion as such. ….when patients get admitted, they come under our care, we’re prescribing their chronic condition medications while they’re there. Again, if they’re on the virtual ward they’re still, by definition, under our care, so we should carry that on until we’re able to discharge them back to the communities, then it’s the GP’s role again.* P16-D

However, other participants believed that a patient’s general practitioner was in a better position to continue to manage their non-COVID chronic conditions due to existing relationships:

*….. my understanding of it was, was for non-COVID related conditions, they could access their GP. For COVID related conditions they would access us … [who] do have a chronic condition and want to see their GP for a very well-known condition or established relationship, it was important for that to be allowed as part of the process. But obviously, being an inpatient and seeing your GP, that’s the difficult part*. P8-N

The question as to who was responsible for the management of chronic conditions was particularly challenging considering cases where patients were on medicines with extra regulatory requirements. One of the participants described a scenario where there was uncertainty of who was supposed to prescribe medicine for a patient with chronic pain:

*I got contacted by [a pharmacist] in our community clinic because there was a palliative patient … on methadone tablets for chronic pain and had been for some time. Something happened in the GP surgery, it was a new GP and the new GP said, ‘No, I can’t prescribe methadone’. … they wouldn’t prescribe it. So [it] would be that virtual ward doctors [who] will take ownership and prescribe it. … But they refused to because they thought that that wasn’t their responsibility. It’s like defining who is responsible for these patients sometimes can be helpful, especially when there’s like tricky things like methadone that need to be prescribed.* P6-P

### Communication challenges when pharmacists work remotely

All participants commented that medication management was challenging due to pharmacists not being present at COVID-19 IPUs. Pharmacist participants described the difficulties experienced the not being able to communicate with patients face-to-face. Pharmacists reported on difficulties with needing to contact patients via phone calls as patients would often not answer their phones, potentially due to not knowing it was a hospital staff member calling:

*… I couldn’t get a hold of the patient after multiple attempts, I would ask [the nurse] to see if their phone was working or get the patient to call me back or to even hand their phone to the patient to chat to me, or whatever it might have been.* P6-P

Providing medicine counselling over the phone was problematic as assessment of a patient’s understanding was difficult due to the absence of non-verbal cues.

*When you're in front of a patient, you can see if [they are] understanding what you're saying. And obviously you've got a medication list in front of, you’ve got the physical meds so it’s a lot easier to counsel and to see if they understand. But when you’re giving someone counselling over the phone, you’ve got nothing. Essentially, you’re just hoping that they can obviously read the medication summary and take the meds appropriately. But you really have no idea.* P14-P

Various participants suggested that video calls to patients would have been a better option, for example through use of the patient entertainment system already in place:

*… so that they could actually do a video into the patient's room. So that would have been even better because they could physically see the patient. So if we had a pharmacist on the ward, they could have sat in one of the doctor's rooms and did a conference call like that. … That would have been a lot better as well. It might have fast tracked a lot of things and a lot of issues that we had during COVID*. P9,10—N

Nurse participants explained the extra pressures placed on nursing staff as they had to perform tasks that would usually be done by IPU pharmacists, which added significant workload. These included taking medication histories and check medicine brought in by patients. This was complicated by the need ‘*to minimise entering patients’ rooms to provide bundles our care.’* P12-N. This caused delays in being able to obtain and provide medicine information which subsequently impacted patient transfers*.* Provision of verbal medicine information to patients was challenging when the nurses were not familiar with the medicines, and in those instances the nurses had to phone pharmacists for advice. Nurse participants also provided examples of increased error and patient safety risks:

*There was a couple of cases where medications got brought up and it was the wrong patient’s label on the front of the bag. So you would have to physically make sure that you checked all those boxes and all those bottles that were in the bags. I found myself doing that. … So you check everything. So that was time consuming. Whereas a pharmacist would normally do that with the patient before sending them home.* P9,10-N

Nurses had to chase after doctors on behalf of pharmacists when pharmacists were not able to contact the doctors via phone calls, e.g. to correct errors on prescriptions. The absence of pharmacists at IPUs caused delays in patient transfers as it was challenging to communicate with doctors over the phone to do the medicine reconciliation and generate prescriptions. Delays resulted in extra pressure on the hospital dispensary as transfers were only sorted out late afternoon.

In addition to the reduced pharmacist support available to do medicine reconciliation, the doctor participants also explained that they found it particularly challenging to prescribe new antiviral medicines. Presence of an IPU pharmacist could potentially have saved the doctors time and taken some of the load off them in obtaining up-to-date evidence-based information on the new therapies, especially as some of the new therapies required consideration of prescribing restrictions, patient criteria, contraindications and potential drug interactions:

*[not having pharmacist on IPU] made things more difficult because we were dealing with a lot of very unfamiliar medications, and it was actually on a daily basis that the stipulations of these medications were changing and it was extremely difficult to keep abreast of what these changes were. …. [It would have helped] enormously [to have a pharmacist on the ward] …. it would have saved potentially hours every day.* P18-D

### Proactive management of specific medication safety risks

The high volume of patients that had to be transferred to virtual models of care within short timeframes required prioritisation of patients who were most at risk of potential medication-related harm. However, participants described the difficulties involved in prioritising patients for medication reviews due to pharmacists not physically present at IPUs and some patients having specific medication management requirements. Patients who were flagged were those with polypharmacy (> 5 medicines) and on high-risk medicines such as those:

- On insulin who required close monitoring of their blood sugar levels:
  *We’ve had patients who were concerned their blood sugars are high, who haven’t got a ketometre or their pump.* P5-D
- Who were newly prescribed an opioid with risk of oversedation:
  *we started an opioid, there would be risk of oversedation or medication harm*. P19-D
- Swapping from one anticoagulant to another anticoagulant:
  *I had a patient that had changed from rivaroxaban to apixaban. … Because of being a high-risk medication, the last thing you want is for them to not take the anticoagulant properly or to double dose or anything like that.* P6-P
- Who received the new antivirals which required monitoring:
  *The potential for interaction harm seems to be quite large with the drug interactions, but also ensuring the appropriate counselling about the adverse effects and making sure they're using the appropriate contraception because of the unknown risks to babies…, and breastfeeding. P6-P*

Pharmacist participants provided examples of scenarios when they were concerned about a patient’s medication safety and approaches followed used to address those concerns. These included asking nurses to go through the patient’s DMR with the patient, making phone calls to community pharmacies to organise dose administration aids, obtaining permission from patients to talk to a family member and requesting doctors to delay a patient’s transfer:

*… we didn't feel that it was safe because of the conversation that [other pharmacist] had with the patient where they didn’t seem to understand their medications. There were multiple changes, they wanted to go home, but I didn’t think it was appropriate at that time. … by just being involved in the discharge process, we could say, ‘No, we don't think they're safe. Can we wait until tomorrow so we can organise something with the community pharmacy?’* P6-P

Nurse participants had concerns about the increased risk of medication administration errors as it was not possible to follow normal procedures:

*You don’t have the computer right beside you to confirm it, double check it, being able to scan the patient’s ID bracelet to positively identify them, that we wouldn’t always do with their medication, as a first point of safety is gone. But there’s no real workaround for that*. P12-N

The risk of medication administration errors was augmented by pharmacists not being able to check patients’ bedside medicines, as is usual practice when pharmacists are present at IPUs.

## Discussion

This study provided insights into clinicians’ experiences and perspectives on the medication management of COVID-19 patients who transferred from an IPU to a virtual model of care. Although clinicians were overall supportive of the virtual models, patient safety issues were raised that will need to be addressed in the development of future services. Through thematic analysis four main themes were identified that highlighted to medicine management challenges involved in the handover to virtual care that potentially caused patient safety risks. The results may be used to inform improvements in medication management strategies of future similar service models.

Overall, clinicians were supportive of the virtual models of care however all participants commented on challenges with pharmacists not having a physical presence on IPUs. Remote service provision impacted on pharmacists’ workload due to communication challenges with the other clinicians as well as with patients. Doctors and nurses highlighted a specific need for support to prescribe and administer the range of therapies to manage COVID-19 that became available at the time of the surge. These therapies have multiple precautions/contraindications, require dosage adjustments for patients with renal or liver impairment, and have potential interactions [37–39]. Clinicians were unfamiliar with these medicines at the time of the surge which subsequently resulted in challenges with medication management of patients. Participants also highlighted safety concerns with patients and specific high-risk medicines that require additional monitoring and therefore prioritisation for pharmacist review [40]. A study conducted in Malaysia with hospitalised older adults with COVID-19 showed 191 of 553 patients (335) were on at least one inappropriate medicine, hence highlighting the need for pharmacists to review patients medicines [41]. Strategies suggested to overcome medication management challenges included for pharmacists to be present on IPUs without face-to-face contact with patients to facilitate multidisciplinary communication.

Pharmacists highlighted the challenges in communicating with patients via telephone which included difficulties with medication counselling as not able to see patients. These findings are similar to a study conducted in Netherland that showed both patients and healthcare professionals preferred face-to-face consultations over telemedicine for newly diagnosed patients [42]. Suggestions for future implementation of virtual models included use of video calls. These findings are supported by a UK survey of 866 clinicians that explored the use of telehealth during the COVID-19 pandemic that showed most preferred video over phone when asked to select a modality and a scoping review on virtual care delivery [43, 44]. Pharmacists in our study reported that telephone calls increased their workload due to patients not answering their phones. A survey of Australian outpatient pharmacy departments that explored the use of telepharmacy during the 2020 COVID-19 lockdowns showed telepharmacy services disrupted pharmacists’ workflow and increased workload compared to face-to-face services [45].

A need was identified to better clarify the roles between hospital and primary care clinicians for the management of patients’ chronic conditions. Participants had varied opinions as to whether hospital doctors were responsible for patient monitoring and for providing repeat prescriptions or if this was the responsibility of patients’ GPs. These uncertainties posed a patient safety risk considering that transitions of care are high-risk periods for medication-related harm [22, 46]. Most patients from our study underwent changes to their medicines whilst in hospital [47]. Considering the uncertainty about roles to manage chronic conditions at transfer, patients had potential risk of MRH. Our study highlighted a need for the clarification of roles between hospital and primary care clinicians for the management of patients’ medicines for chronic conditions in virtual models of care. Studies have shown successful implementation of virtual models is feasible and can provide similar outcomes to traditional face-to-face care when there are clear communication pathways between hospital and primary care clinicians [48–51].

### Strengths and limitations

The 20 hospital clinicians interviewed represented doctors, pharmacists and nurses which enabled the research team in obtaining experiences and perspectives across all three professional groups. Interviews were mostly conducted face-to-face which helped to foster a relationship between the interviewer and interviewee, enhancing the depth of information obtained. The methodological approach was structured and transparent with all participants provided an opportunity to review their verbatim interview transcripts.

This research focused on perceptions of clinicians from two Australian public hospitals from one health service and the results may not be transferable to other public hospitals or jurisdictions. The models of care introduced to GCHHS during the COVID-19 surge may not apply to other virtual models of care. Our study focused on medication management aspects during transition from an IPU to a virtual model of care and not on other issues that clinicians could have experienced.

## Conclusion

The COVID-19 surge end of 2021/beginning of 2022 required hospitals to implement innovative models of care. This study explored clinicians’ perceptions about the medication management of patients who transferred to virtual models of care and as such aligned with local priorities aimed at facilitating transitions of care. Clinicians were supportive of the models that were introduced although they identified medicine handover gaps that could have resulted in patient safety risks. The results from this study provide useful information to improve medication safety implementation aspects of future virtual models of care.

## Data Availability

The datasets used and/or analysed during the current study are available from the corresponding author on reasonable request.
